# Investigation on the Performance of Partial Penetration Welds in Multicell Concrete Filled Steel Tubes

**DOI:** 10.3390/ma14247543

**Published:** 2021-12-08

**Authors:** Lian-Jin Bao, Fei-Fei Sun, Osama Mughrabi, Liu-Lian Li, Guo-Qiang Li

**Affiliations:** 1 School of Civil Engineering, Tongji University, Shanghai 200092, China; blj0068@ecadi.com (L.-J.B.); ffsun@tongji.edu.cn (F.-F.S.); gqli@tongji.edu.cn (G.-Q.L.); 2 East China Architectural Design & Research Institute, Shanghai 200002, China; 3 State Key Laboratory of Disaster Reduction in Civil Engineering of China, Tongji University, Shanghai 200092, China; 4 China Construction Second Engineering Bureau Ltd., Beijing 100160, China; 54600942@163.com

**Keywords:** concrete filled steel tubes, partial penetration weld, elephant foot buckling, 2-cell specimens, 4-cell specimens, weld tearing, confinement pressure, regression analysis

## Abstract

This paper presents an experimental and analytical investigation on the performance of partial penetration welds used to adjoin steel plates in irregular shaped multicell concrete filled steel tubes. The experimental program of this study is designed based on an actual implementation of such members as mega columns in a super high rise building in China. A total of six specimens are designed with different plate arrangements for the purpose of testing the performance of the partial penetration welds at different locations of the specimen. The designed specimens are tested under different load procedures and directions; this is achieved by placing them in vertical and slantwise manners between two loading plates which impose monotonic and cyclic actions. The failure conditions of each of the tested specimens are presented and discussed in detail and are based on the conclusions drawn from the experimental observations; the partial penetration weld at the corner of the tested specimens is found to be the most vulnerable. To facilitate large scale analysis, a finite element model constructed by the finite element analysis program ABAQUS is verified against experimental results. The evaluation of the stress at the partial penetration welded corner is carried out following an empirical procedure, which is adopted due to the complexity of the problem domain. The adopted procedure consists of two steps: the first one is to initially evaluate the stress based on an existing method in the literature, and the second one is to fit the results of the initial evaluation with the finite element model results based on parametric and regression analysis. After performing regression analysis, a formula to predict the weld stress is concluded, and the results of the proposed equation are found to be satisfactory when compared with the finite element model results.

## 1. Introduction

Concrete filled steel tubes (CFSTs) are currently gaining more popularity over conventional reinforced concrete columns. The fact that they combine the stoutness of concrete and the ductility of steel makes them a preferable choice, especially in seismic applications, not to mention the enhanced concrete compressive strength due to the confinement effect. Typical CFSTs have single-cell circular or rectangular shaped forms with a variation of sizes depending on the design requirements. However, in this study, the behavior of irregular shaped multicell CFSTs made by welding steel plates is the one to be investigated.

As a first step, it is essential to understand the constitutive behavior of confined concrete since the confinement pressure is expected to be a major contributor to the stress at the welded corner. Studies aiming to develop a stress strain relationship curve for confined concrete are widely available in the literature [1,2,3,4,5,6,7,8]. These studies proposed several constitutive relationships for the confined concrete under different load conditions, different confining materials and for different shapes.

Having relatively thin steel plates as the outer tube confining the inner concrete core, a CFST is vulnerable to typical problems faced in hollow steel tubes under compression such as plate buckling. However, the presence of an inner concrete core prevents inward buckling of the tube plates and restrains them to buckle outwards. Although several studies discussing the overall and local buckling behavior of the steel tube are available, these studies have mainly focused on evaluating the elastic buckling load and the cross-sectional capacity of the member [9,10,11] or the reduction in the cross-sectional capacity [12,13,14].

CFSTs with either full or partial penetration welding details are not comprehensively covered in the literature. In the few available studies, tearing of the welded corner is recorded and visual evidence is provided [15,16]. However, up to date, there are no available studies properly addressing the stresses affecting the welded corner especially due to sever local buckling.

The stresses at the welded corner are a result of the vertical load and the internal confinement pressure taking effect simultaneously. Several attempts were made to establish the governing equations for the buckling shape of steel plates in rectangular members subjected to similar loading conditions as in the specific publication of Timoshenko and Krieger [17], which provides a comprehensive and valuable set of solutions for various plate shapes under various loading conditions. However, for plates under the combination of vertical load and internal pressure, the presented solutions exist only for circular shells, and the task to develop a similar solution for the objective of this study would have been time consuming and far from the main goal. Batikha et al. [18] and J. F. Chen et al. [19] have presented a full set of equations to calculate the stress caused by elephant foot buckling for circular soil tanks subjected to similar load conditions. Aiming to strengthen these structures against elephant foot buckling, their work includes procedures that are valuable for this study due to the similarities in the outward elephant foot shaped buckling of the steel tube in both studies.

In the following analysis and study, the behavior of irregular shaped multicell welded CFSTs is investigated experimentally, implementing partial penetration welds for members subjected to monotonic and cyclic loads. The performance of this specific weld detail under the simultaneous effect of vertical and confinement pressures, which will lead to severe outward local buckling, is experimentally documented for the first time. An equation to calculate the resultant stress at the partial penetration welded corner is also presented, which can be useful for design purposes. In the subsequent sections of this paper, the experimental program and test results associated with this study are first presented. The main observation of the experimental program is the tearing of the partial penetration welded corner of specimens with higher width to thickness ratio near the location of elephant foot buckling. A FEM model constructed by ABAQUS [20] is then verified against experimental results, facilitating a large-scale analysis. Keeping in mind the main experimental observation and aided by the constructed FEM model, a method to initially evaluate the stress at the welded corner is developed in the last section of this paper. This method that is developed for circular cross-sections may be transferred to rectangular forms roughly by assuming that both types of cross-sections have the same load capacity. However, it is not expected that this will yield satisfactory results, and a regression analysis is performed later on to fit the results based on the proposed equation having a wide range of parameters with the results obtained from the analysis using the constructed FEM model.

## 2. Experimental Program

The main objective in the experimental program of this study was to evaluate the feasibility of using partial penetration weld at different locations of multicell CFSTs that have irregular configurations. The requirement to study this type of members was raised from an actual implementation as a mega-column of a high rise building in China. However, due to size limitations, the columns had to be scaled down and new cell configurations were implemented. The new configurations were executed by welding steel plates with different weld details including partial penetration weld to evaluate the performance of this weld detail.

### 2.1. Description of Test Specimens

**Figure 1:**
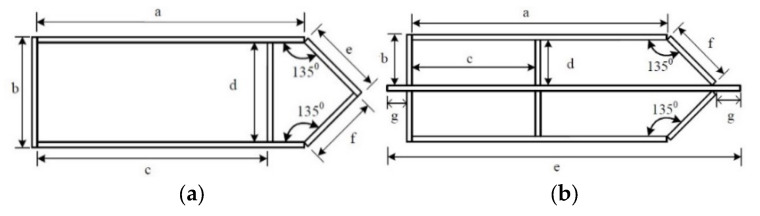
Cross-section shape of the specimens. (**a**) 2-cell specimens; (**b**) 4-cell specimens.

**Figure 2:**
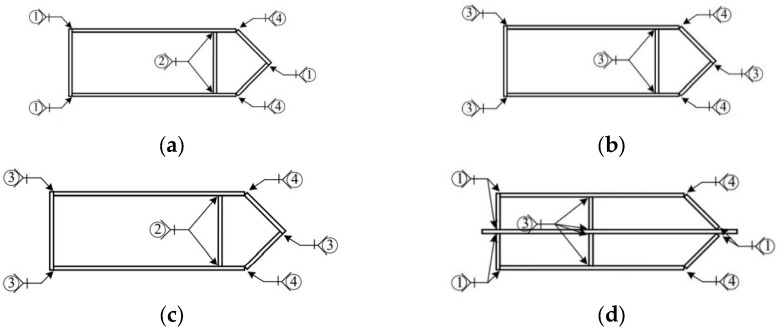

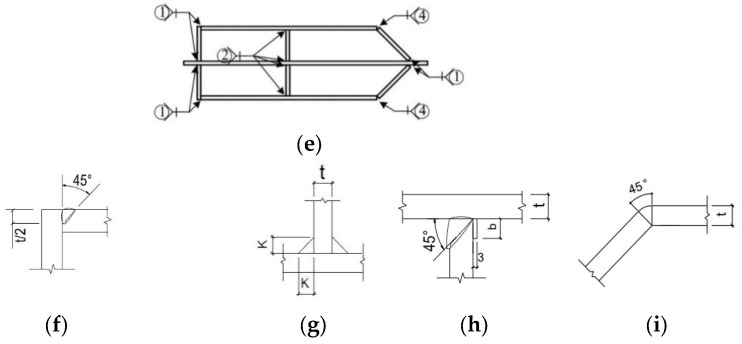
Weld locations and details. (**a**) PEN1 and PEN3; (**b**) PEN2; (**c**) PEN4; (**d**) PEN5; (**e**) PEN6; (**f**) Weld detail 1; (**g**) Weld detail 2; (**h**) Weld detail 3; (**i**) Weld detail 4.

**Figure 3:**
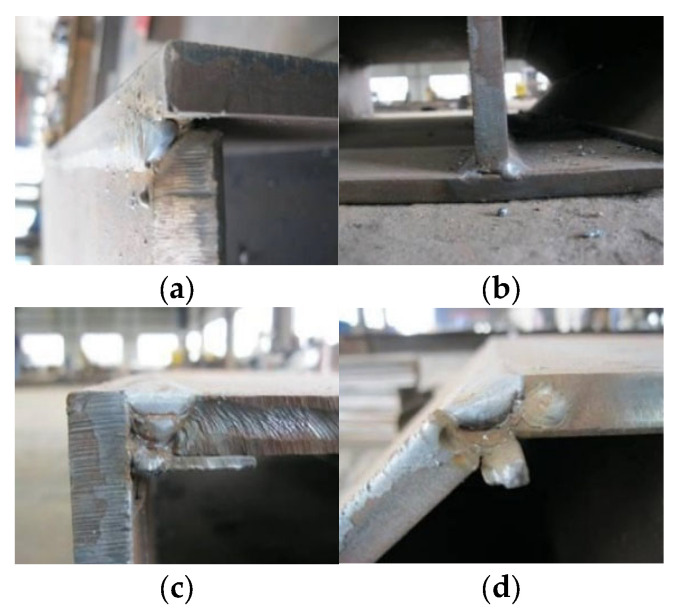
Weld details before casting of concrete. (**a**) Weld detail 1; (**b**) Weld detail 2; (**c**) Weld detail 3; (**d**) Weld detail 4.

A total of six test specimens, labelled as PEN1 to PEN6, were fabricated, and they can be categorized into 2-cell and 4-cell groups, as shown in Figure 1. Four of the fabricated specimens were of the 2-cell group while the other two specimens were of the 4-cell group, with dimensions as reported in Table 1. The specimens were made by welding 14 mm thick steel plates using different weld details at different locations, and the location of each weld detail used is shown in Figure 2. The different weld details were executed using a gas metal arch welding process with CHW-50C6SM welding consumable, and the final weld details before casting concrete are shown in Figure 3.

**Figure 4:**
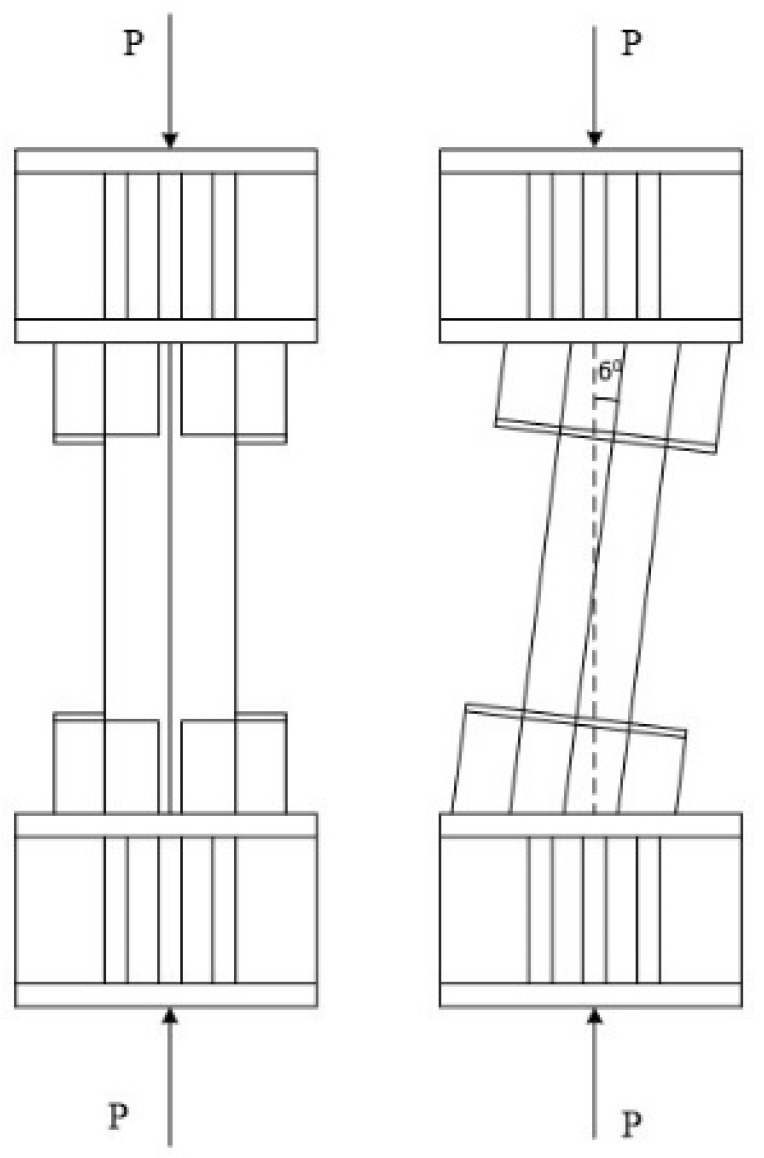
Vertical and slantwise positioning of the specimens.

Cyclic loading procedure was applied to specimens PEN1 and PEN4 to PEN6, whereas specimens PEN2 and PEN3 were tested using monotonic loading. For developing a combination of axial, shear and bending action effects, specimens PEN2 to PEN6 were placed in a slantwise position between the loading plates, as shown in Figure 4. The net height of all specimens was 1000 mm, and after adding the stiffening connection plates at both ends, the total height became 2520 mm.

### 2.2. Material Properties

The fabricated outer tube was made of Q235B grade steel, which, based on the Chinese code GB/T 700, has a yield strength of at least 235 MPa and an ultimate strength of 370 to 500 MPa, and the target infill concrete strength was 35 MPa for all specimens. To confirm the compatibility of the used materials with the selected standards, the properties of these materials were tested as reported in Table 2 and Table 3. The material of the outer steel tube was tested in room temperature based on the Chinese code GB/T228-2002, which is applicable to determine the tensile performance of metallic materials at ambient temperature. The samples were taken from two different steel plates according to the Chinese code GB/T2975-1998, which specifies sampling location and sample preparation for mechanical testing of metallic materials. Six samples were tested in total, and the results are reported in Table 2. For the concrete properties, a total of nine 150 mm cubic specimens were tested according to the Chinese code GB50010-2002 at the age of 28 days, and the results are shown in Table 3.

### 2.3. Employment of Displacement and Strain Gauges

**Figure 5:**
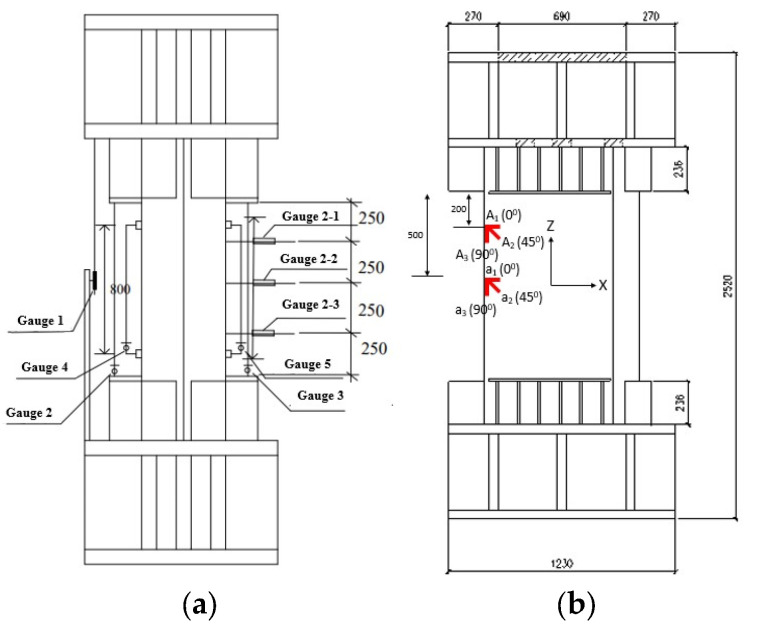
Locations of displacement and strain gauges. (**a**) Displacement gauges; (**b**) Strain gauges near the welded corner.

Displacement and strain gauges were distributed in several locations around the perimeter of each test specimen. Figure 5a shows the distribution of displacement gauges along the height of the specimen, which had a range limit of 100 mm and an accuracy of 0.02 mm. For the sake of not complicating the content of this paper, only strain gauges installed near the weld location along the height of the specimen are shown in Figure 5b. The strain gages were installed for measuring the circumferential, 45° inclined and vertical strains for each of the two points shown in Figure 5b.

In Figure 5a, a total of eight displacement gauges are shown, and they can be divided into two groups, the first of which was aimed for measuring the axial displacement so that gauge 1 was used to measure the relative displacement between the endplates, gauges 2 and 3 were used to measure the displacement relative to the mid-cross-section within a monitoring length of 1000 mm and gauges 4 and 5 were used to measure the displacement relative to the mid-cross-section within a monitoring length of 800 mm. The second group contains gauges 2–1, 2–2 and 2–3, which were used to measure the lateral displacement at different locations along the column heights. The angle values shown in Figure 5b refer to the angle between *X*-axis and the measurement direction of the strain gauge.

### 2.4. Overall Behavior of the Tested Specimens

#### 2.4.1. Failure Condition

**Figure 6:**
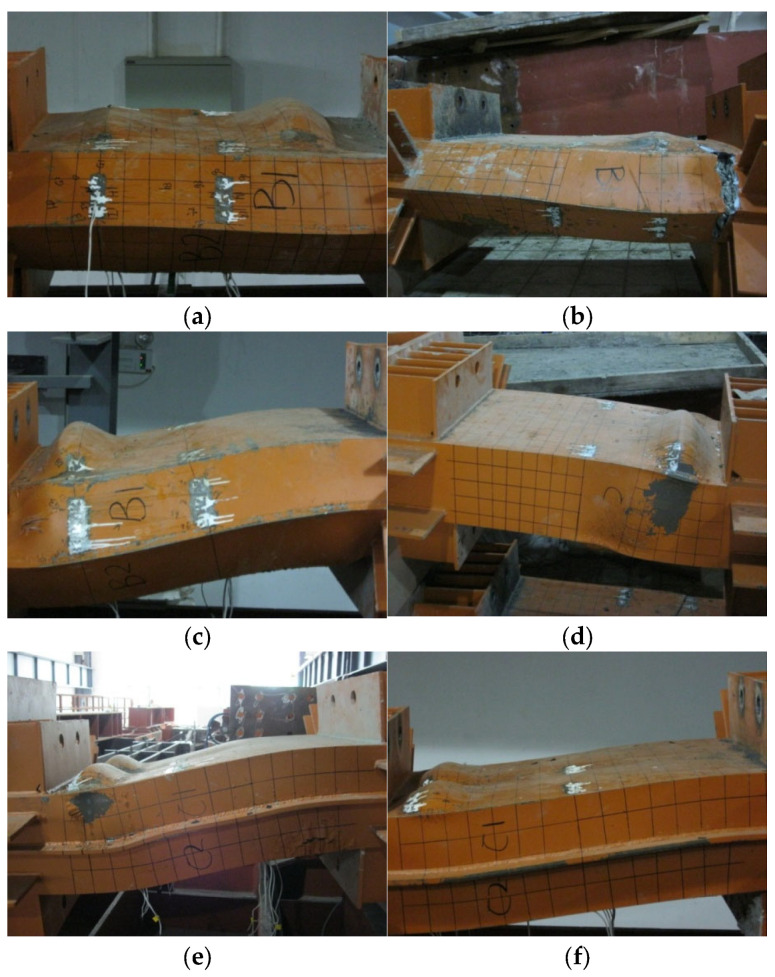
Failure modes of test specimens. (**a**) PEN1; (**b**) PEN2; (**c**) PEN3; (**d**) PEN4; (**e**) PEN5; (**f**) PEN6.

Failure loads of the tested specimens are reported in Table 4; the compression, shear and bending components of the applied load can be calculated by referring to Figure 4. Moreover, the failure conditions of the tested specimens are shown separately in Figure 6a–f.

The most distinct feature of the tested specimens is the formation of large outward buckling in the steel tube near the end support, and this type of failure is referred to as an elephant foot buckling due to its shape and location. The elephant foot formed in the case of 2-cell specimens is bigger than the one formed in 4-cell specimens, which was also located closer to the end support. The average location of the peak elephant foot buckling for the 2-cell specimens is around 150 mm from the end support, while the average of the same distance is around 110 mm for the 4-cell specimens. The steel tube of the tested specimens remained intact except for specimen PEN2, which experienced tearing in the steel tube near the base.

**Figure 7:**
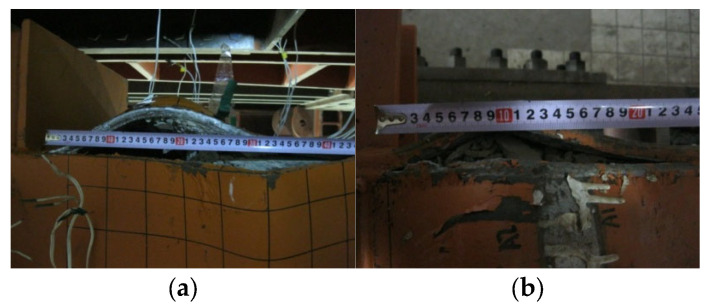
Tearing of the weld in the 2-cells specimens with partial penetration welds. (**a**) PEN1; (**b**) PEN3.

The main observation concerning the welded corner was tearing of the weld near the location of the elephant foot buckling in the 2-cell partial penetration welded specimens, PEN1 and PEN3, as shown in Figure 7. The performance of partial penetration weld is expected to be inferior when compared with other weld details. However, the same weld detail remained intact in the case of 4-cell specimens, and this observation will be illustrated further in the proceeding subsections.

#### 2.4.2. Influence of Cyclic and Monotonic Loading Procedures

**Figure 8:**
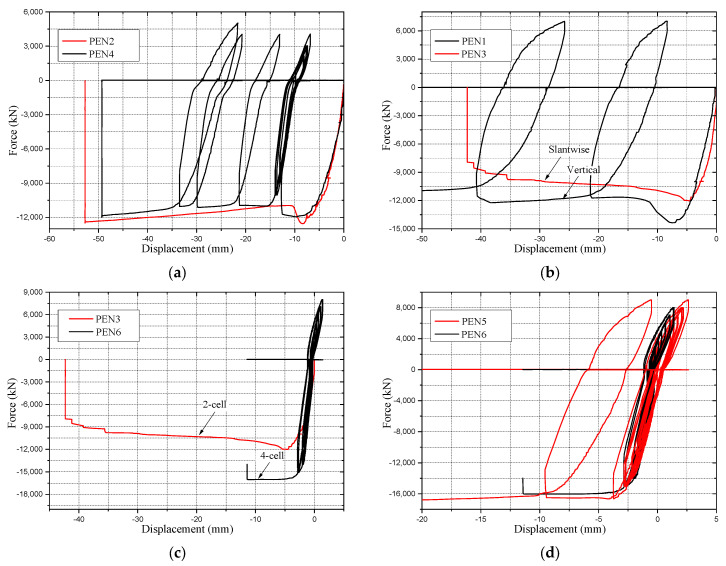

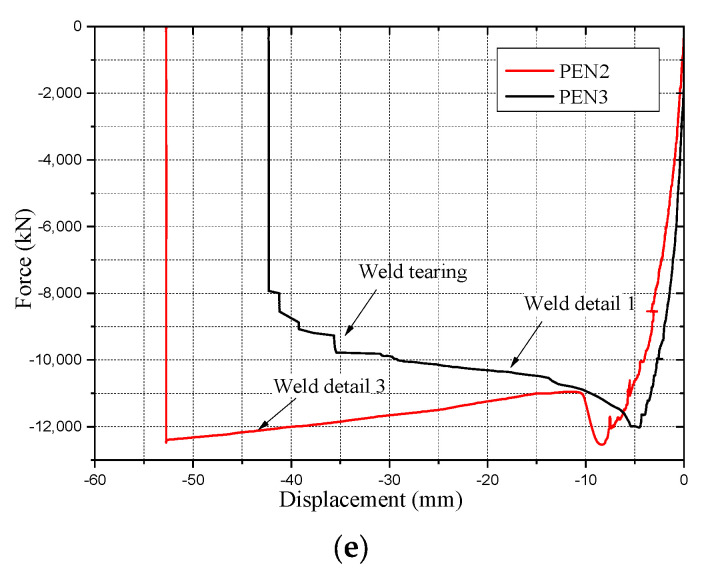
Axial performance of the tested specimens with different parameters. (**a**) Cyclic and monotonic loaded specimens; (**b**) Vertical and slantwise loaded specimens; (**c**) 2-cell and 4-cell specimens; (**d**) Samples with different internal weld details; (**e**) Samples with different corner weld details.

The influence of loading procedure is shown in Figure 8a, where the force-displacement curves of specimens PEN2 and PEN4 are plotted together. Both specimens had the same parameters, except for the internal weld detail, which is later shown to have a slight influence on the behavior of the specimen. As shown in the figure, the loading procedure had only a slight influence on the performance of the tested specimens, since the compressive strength of the concrete infill in both specimens was almost the same according to the material tests, and both specimens showed a similar ultimate response when comparing the plotted force displacement curves that have a difference not greater than 4.5% in their maximum cross-sectional capacities.

#### 2.4.3. Influence of Vertical and Slantwise Load Orientations

The load orientation is expected to have a significant influence on the performance of the tested specimens, simply because a slantwise specimen will be subjected to a combination of axial, shear and bending actions, which will lead to a lower axial failure load than vertically positioned specimens. To detect the influence of load orientation, the force-displacement curve of specimen PEN1, loaded vertically, and specimen PEN3, loaded slantwise, are plotted together in Figure 8b. Apart from the loading procedure that was concluded to have only a slight influence, both specimens had similar parameters. In this case, the difference in the recorded maximum cross-sectional capacity is significant as compared to the influence of changing other parameter values: the maximum capacity for specimen PEN1 was 13,992 kN and for specimen PEN3 11,980 kN, indicating a difference of almost 17%.

#### 2.4.4. Influence of Cell Number (2-Cell vs. 4-Cell Specimens)

Both 2-cell and 4-cell specimens have almost the same cross-sectional area. However, the 4-cell specimens had significantly lower plate width to thickness ratio, which is expected to influence their performance. Figure 8c shows the force-displacement curves of specimens PEN3 and PEN6 plotted together. Although the two specimens had identical parameters except for the number of cells, specimen PEN6 had a much higher maximum cross-sectional capacity than specimen PEN3. The recorded maximum capacity was 11,980 kN for specimen PEN3 and 15,838 kN for specimen PEN6, i.e., the difference is 32%.

### 2.5. Performance of Specimens with Different Weld Details

#### 2.5.1. Influence of Weld Detail in Internal Connections

For detecting the influence of weld detail in internal connections, the plots of force-displacement curves in Figure 8d for the PEN5 and PEN6 specimens are compared. Both specimens had identical parameters, except in the internal weld connections. As shown in Figure 8d, the response of both specimens was almost similar, with a slight difference due to the higher compressive strength of concrete in PEN5, as shown in Table 3. The maximum load capacity for specimen PEN5 was 16,023 kN and for specimen PEN6 15,338 kN, i.e., the difference is only 4.5%.

#### 2.5.2. Influence of Corner Weld Details

The influence of corner weld detail is shown in Figure 8e, where the force-displacement curves of specimens PEN2 and PEN3 are plotted together. The only difference between both specimens besides the corner weld detail was the internal weld detail, which was concluded to have a slight influence. The difference in axial stiffness of both specimens before reaching the ultimate capacity of the cross-section was more prominent compared to the influence of welds in the internal connections and keeping in mind that both specimens were fabricated using the same batch of concrete. The maximum cross-sectional capacity is not significantly influenced, with a difference of 4.7% between both specimens. Tearing of the partial penetration welded corner is shown in the form of a sudden drop in the force-displacement curve that happened in the late plastic stage.

**Figure 9:**
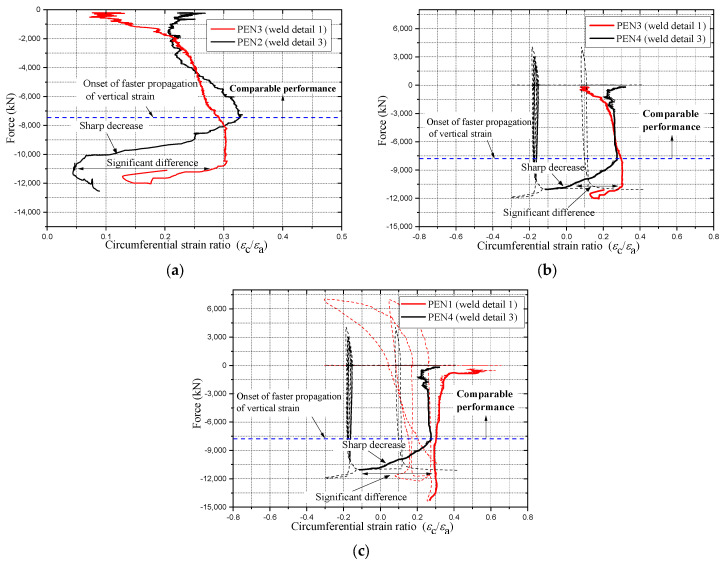
Circumferential strain ratio at the welded corner for different weld details. (**a**) PEN2 vs. PEN3; (**b**) PEN3 vs. PEN4; (**c**) PEN1 vs. PEN4.

A deeper look at the recorded strains near the welded corner is necessary to further understand the behavior of partial penetration weld detail; this is carried out by plotting the circumferential strain ratio of the recorded strains near the corner for different weld details, as shown in Figure 9a–c. The circumferential strain ratio is obtained by dividing the circumferential strain ($\varepsilon_{c}$) over the axial strain ($\varepsilon_{a}$).

Based on the results presented in the diagram of Figure 9, the plotted performance can be divided into two stages. The first stage is one at which the performance of both weld detail 1 (partial penetration) and weld detail 3 (full penetration supported with a backplate) is comparable; at this stage the development of vertical and circumferential strain is almost equal, and no sudden changes in the pattern of the curves appeared. This comparable performance continued until the applied load reached around 60% of the cross-sectional capacity. In the second stage, and after the applied load exceeded 60% of the cross-sectional capacity, specimens with weld detail 1 at their corners started to experience a sharp decrease in the circumferential strain ratio, as shown in the diagrams. On the other hand, no significant change in the circumferential strain ratio was observed for weld detail 3, creating a significant gap at the end of the elastic state. The difference in the performance between both weld details at the second stage indicates an inferior performance of weld detail 1, which can make it more vulnerable to tearing, especially in the case of an existing weld crack caused by the rapid heating and cooling of the metal around the weld location.

#### 2.5.3. Influence of Width to Thickness Ratio on the Weld Performance

As shown in Figure 7, the 2-cell specimens PEN1 and PEN3 with partial penetration welded corner experienced weld tearing near the peak height of elephant foot buckling. However, the same weld detail remained intact in the 4-cell specimens PEN5 and PEN6, and this observation, accompanied with the conclusion drawn from Figure 8c, suggests that the weld behavior is dependent on the width to thickness ratio of the plate. In the 2-cell specimens, the width to thickness ratio of the long plate was 41.9, whereas in the 4-cell specimens, the ratio was 23.5.

## 3. FEM Modeling

### 3.1. General

**Figure 10:**
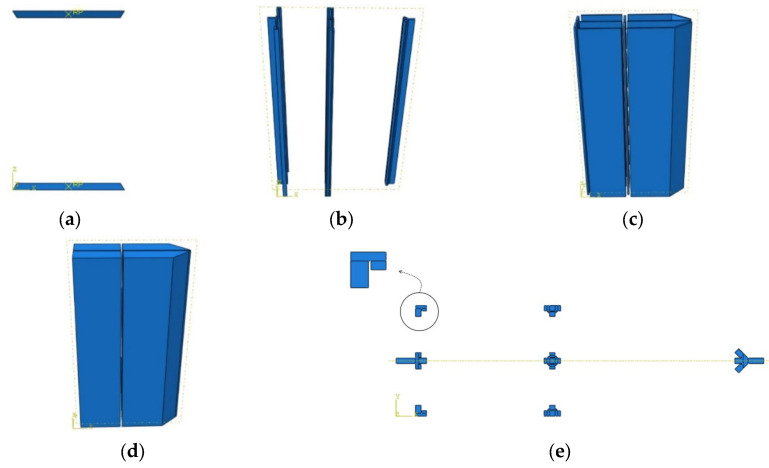
Parts of the FEM model and weld details (PEN5 and PEN6). (**a**) Loading plates; (**b**) Welded corners; (**c**) Plates of the steel tube; (**d**) Concrete core; (**e**) Weld details.

FEM modelling of the test specimens was carried out using the FEM program ABAQUS [20]. Each of the simulated specimens consisted of four components modelled as separate parts, loading plates, concrete core, plates of the steel tube and welded corners, as shown in Figure 10a–d. The loading plates were modelled as discrete rigid shells, while the other parts were modelled as deformable extrusion parts.

**Figure 11:**
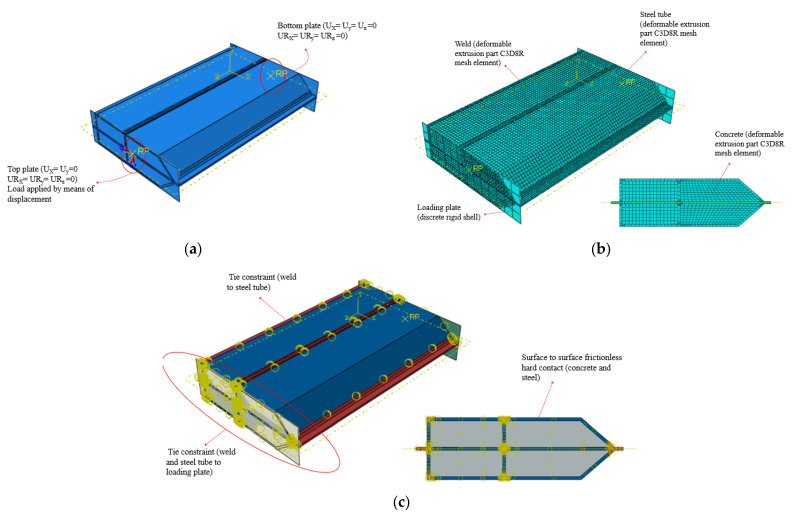
Detailed depiction of the constructed FEM model (PEN5 and PEN6). (**a**) Boundary conditions; (**b**) Meshing; (**c**) Interactions.

All the simulated specimens were assumed to have partial penetration welded corners similar to specimens PEN1, PEN3, PEN5 and PEN6. The partial penetration weld detail at the corners was simulated as a 2 mm gap between welded plates as shown in Figure 10e. The partial penetration weld detail at the corners was simulated as a 2 mm gap between welded plates, as shown in Figure 10e. The 2 mm gap was selected based on the guidelines of BS EN ISO 5817 [21], which allows a maximum of 2 mm gap for stringent quality level; this was also the value maintained while fabricating the test specimens. All parts of the model except the loading plates were modelled using 8-node linear brick (C3D8R) mesh elements, and the mesh was further refined at the weld part where the stresses were to be investigated. The end sections of the column were tied to the loading plates using tie constraints, which were also used to tie the weld to the steel tube. Moreover, the contact between steel and concrete surfaces was defined as frictionless hard contact. A detailed depiction of the constructed FEM model is shown in Figure 11.

### 3.2. Selection of Step Type in Abaqus

The ABAQUS [20] solver provides solutions following two procedures: implicit and explicit procedures. The first trials to model the specimens of this study were carried out using the implicit procedure. However, several nonlinearities had to be accounted for to guarantee accurate results. Nonlinearities including geometrical nonlinearities, contact between the inner steel surface and the concrete core and material nonlinearities had to be defined before running the solver; these nonlinearities and the implementation of solid extrusion parts as the outer steel tube prevented the model from converging using this procedure. To overcome these issues, trials using the explicit procedure, which determines the solution by explicitly advancing the kinematic state from the end of the previous increment [20], were performed. When the explicit procedure was implemented, convergence of the modeled specimens was achieved, with a significantly reduced calculation time [22,23].

The explicit procedure provided by ABAQUS [20] is normally used to solve dynamic problems where the load is applied over a short time period. The loading rate and mass scaling factor, which are normally used to reduce the calculation time, have significant influence on the accuracy and stability of the results; thus, they must be controlled with special attention, which is further explained in the proceeding sections.

### 3.3. Material Properties

#### 3.3.1. Modelling of the Confined Concrete

The confinement effect of the surrounding steel tube on the concrete core of CFSTs has been studied and properly documented by several researchers in this field. It is widely agreed that the confinement effect will enhance the compressive strength significantly in the case of circular CFSTs. However, square CFSTs experience lower enhancement of the compressive strength of the concrete core, and it is often ignored when calculating the capacity of the cross-section. An example of this practice is the equations shown in the guidelines of the European code EC4 [24]. For this study, the confinement pressure of the concrete core against the inner surface of the steel tube is a major contributor to the stresses at the welded corner, which makes it essential to find a model capable of providing acceptable estimations. The model used by Thai et al. [6] was adopted for this study, since the model is well presented in the reference document, and is explained next.

In the elastic state of stresses, the constitutive relationship of concrete is mainly described by the peak stress, peak strain and modulus of elasticity. The peak strain of unconfined concrete can be calculated from Equation (1) [25].

(1) $$\varepsilon_{c}=\left(-0.067f_{c}{}^{2}+29.9f_{c}+1053\right)\times 10^{-6}$$

where $f_{c}$ is the peak compressive strength of unconfined concrete. The concrete modulus of elasticity $E_{c}$ can be calculated according to ACI 318 [26] using Equation (2).

(2) $$E_{c}=4700\sqrt{f_{c}}\left(\mathrm{MPa}\right)$$

The peak stress and the corresponding strain for the confined concrete are then calculated as shown in Equations (3) and (4) [27].

(3) $$\frac{f_{0}}{f_{c}}=1+3.24{\left(\frac{f_{r}}{f_{c}}\right)}^{0.8}$$

(4) $$\frac{\varepsilon_{0}}{\varepsilon_{c}}=1+17.4{\left(\frac{f_{r}}{f_{c}}\right)}^{1.06}$$

where $\varepsilon_{0}$ is the peak strain of confined concrete, $f_{0}$ is the peak stress of the confined concrete and $f_{r}$ is the confinement pressure calculated from Equation (5) [6].

(5a) $$f_{r}=\frac{\left(195.118+40.611f_{y}\right)e^{-0.1\frac{B}{t}}}{988-0.01962f_{c}}\mathrm{for}\frac{B}{t}\leq 15$$

(5b) $$f_{r}=\frac{\left(-42428+236f_{y}\right)e^{-0.04\frac{B}{t}}}{7773+f_{c}^{1.6}}\mathrm{for}\frac{B}{t}>15$$

where $f_{y}$ is the steel yield strength, B is the equivalent plate width and t is the plate thickness. The pre-peak state of the constitutive relationship is calculated as reported in Equation (6) [28,29].

(6) $$\frac{f}{f_{0}}=\frac{\left(\varepsilon /\varepsilon_{0}\right)r}{r-1+{\left(\varepsilon /\varepsilon_{0}\right)}^{r}},\;r=\frac{E_{c}}{E_{c}-\left(f_{o}/\varepsilon_{0}\right)}.\;$$

The equation proposed by B. Binici [30] is used as the constitutive relationship in the plastic state and is given as Equation (7)

(7) $$f=f_{r}+\left(f_{c}-f_{r}\right)\exp\left[-{\left(\frac{\varepsilon -\varepsilon_{0}}{\alpha }\right)}^{\beta }\right]$$

where β is 0.92 and α can be calculated from Equation (8) [31].

(8) α=0.005+0.0075ξ

At which ξ is the confinement coefficient. The above set of equations was implemented in the concrete damage plasticity model provided by ABAQUS [20], and this should properly describe the plastic behavior of the concrete core and at the same time account for the confinement effect.

#### 3.3.2. Modelling of the Steel Tube and Welds

Both the steel tube and the welds were modelled following an elastic-linear plastic model, where the Young’s modulus of steel and Poisson’s ratio were taken as 200,000 MPa and 0.3, respectively. The yield and ultimate strengths of the material of the appropriate test were applied as reported in Table 2, and, for the welded joints, as the values recommended by the manufacturer.

### 3.4. Loading and Boundary Conditions

Loading plates were tied to both ends of the specimen using tie constraints, a reference point was defined at the middle of both plates as shown in Figure 10a and the boundary conditions were applied at these points. The modelled specimens had all the degrees of freedom fixed at the bottom, while at the top, vertical movement was allowed and the load was applied at this point by means of displacement.

As stated earlier, in explicit modelling, special attention has to be paid to the loading rate and mass scaling factor, and several simulations were therefore performed considering different load rates and mass scaling factors. The force-displacement curves of these simulations were compared with the ones from the experiment. The best results were obtained when the rate of loading was maintained at 1 mm/s and the mass scaling factor at 10, which is also the same value of mass scaling factor considered by M.F. Hassanein et al. [22]. For slantwise specimens, a local coordinate system was defined at the top reference point and the displacement load was applied accordingly.

### 3.5. Modelling of Residual Stresses

**Figure 12:**
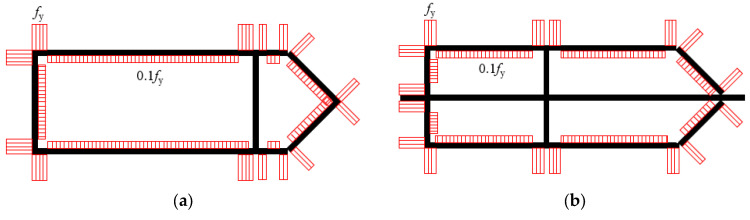
Distribution of residual stresses. (**a**) 2-cell specimens; (**b**) 4-cell specimens.

The residual stresses caused by rapid heating and cooling of the metal during the welding process were accounted for in this study, as recommended by Thai et al. [6], assuming a level of compressive residual stresses equal to 10% of the yield strength. Since the modelled specimens are closer to a steel box in shape, the distribution of residual stresses in this case was based on the ideal distribution in a steel box. The distribution of residual stresses in the 2-cell and 4-cell specimens is shown in Figure 12. The residual stresses were considered in the FEM model as an initial condition using the initial condition option available in ABAQUS [20].

### 3.6. FEM Results and Verfication

Four specimens out of the six ones tested, having the partial penetration welds, were simulated. The FEM results and their verification against experimental data are presented and discussed. The main focus in the comparison is to verify the capability of the FEM model to follow the envelope of the force-displacement curves of the experimental results, as well as the buckling shapes observed in the test.

#### 3.6.1. Force-Displacement Curves

**Figure 13:**
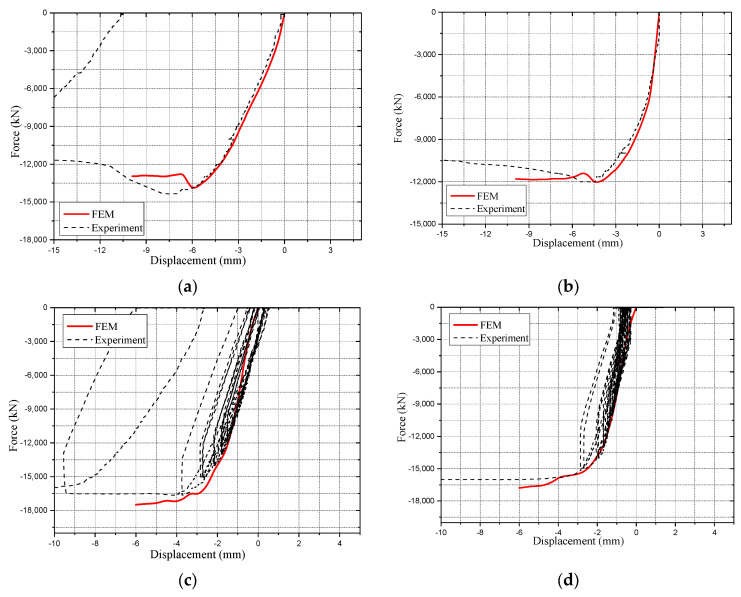
Comparison between the FEM results and the experimental ones. (**a**) PEN1; (**b**) PEN3; (**c**) PEN5; (**d**) PEN6.

The force-displacement curves of the FEM models are plotted together with the ones from the experiment in Figure 13, and it is seen that the FEM models were capable of following the envelops of the experimental curves with an acceptable accuracy. For further comparison between the FEM results and the experimental data, the ultimate capacity loads and the displacement data when reaching them are listed in Table 5.

#### 3.6.2. Buckling Shapes

**Figure 14:**
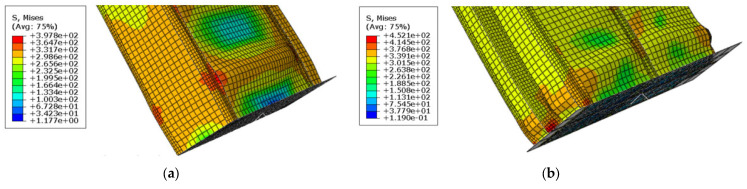
Buckling shapes by the FEM model. (**a**) 2-cell specimens; (**b**) 4-cell specimens.

In both 2- and 4-cell specimens, an elephant foot buckling was initiated near the support end. Due to the smaller size of plates in the 4-cell specimens, smaller elephant foot buckling developed in them as compared to the 2-cell specimens. The shapes and locations of the buckling formed in the FEM analysis are shown in Figure 14.

The agreement between the experimental results and the FEM models results regarding the shapes and locations of buckling is also acceptable, e.g., the FEM modelling for the PEN3 specimen produced the formation of the peak in the elephant foot buckling 163 mm from the base whereas the respective distance of 150 mm was observed in the test. For the PEN5 specimen, the respective location evaluated by FEM was 140 mm and 110 mm, as observed in the test.

## 4. Evaluation of the Resultant Stress at the Corners with Partial Penetration Welds

### 4.1. General

**Figure 15:**
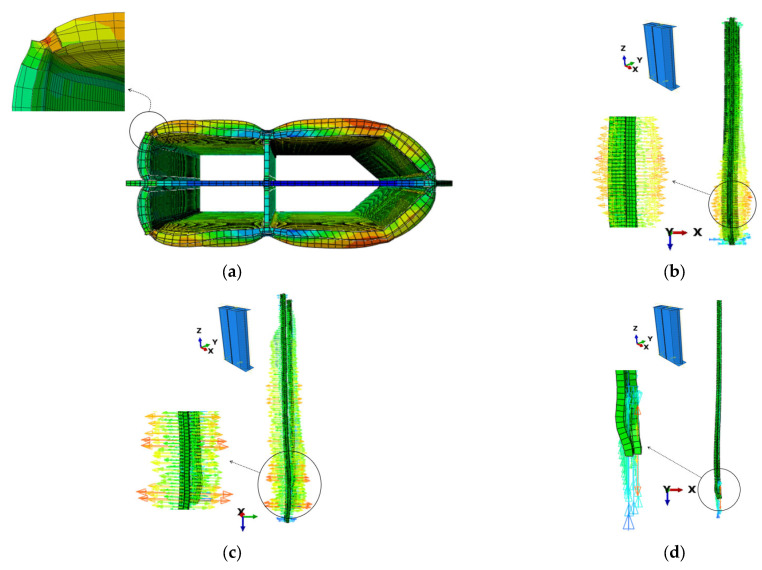
The complexity of the stress state at the welded corner. (**a**) Section view of the partial penetration welds after the initiation of the elephant foot buckling; (**b**) Circumferential stress component (S11); (**c**) Lateral stress component (S22); (**d**) Vertical stress component (S33).

The feasibility of using partial penetration welds to fabricate the external steel tube of welded CFSTs was experimentally evaluated, and based on the test results, the use of partial penetration welds is only feasible for internal connections or when the plate width to thickness ratio is small enough. For other cases, proper evaluation of the stresses at the welded corners is required. An illustration of the complexity of the stress states at the partial penetration welded corner in the three principal planes is shown Figure 15.

Figure 15 shows severe irregularity in the deformed shape of the welded corner. Moreover, the circumferential stress component S11 and the lateral component S22 are nonuniform, especially around the location of the elephant foot buckling. Since these fluctuations are directly related to the formation of the elephant foot buckling, finding a theoretical background to evaluate the stress proved to be challenging; for this reason, an empirical approach is adopted for this study. The adopted approach relies on fitting with the results of a large number of simulations performed using the constructed FEM model.

The first step in the adopted empirical approach is to roughly evaluate the stress components at the welded corner using one of the available theoretical procedures in the literature; for this, the procedure to calculate the stresses caused by elephant foot buckling for cylindrical plates presented by Batikha et al. [18] is implemented. The second step is to combine the calculated stress components in a yield criterion, which is achieved by implementing the yield criterion presented in the European code EC3 [32] for fillet welds. The method presented in the European code EC3 [32] is designated for fillet welds and considers the weld throat plane as the critical plane. However, in the case of this study, the location of the critical plane is unknown. The difference in the shape of the cross-sections between the procedure of Batikha et al. [18] and this study, as well as the unknown location of the critical plane of the weld, means that the calculated stress resultant would be unsatisfactory. However, regression analysis is later used to fit the results of the adopted approach with the FEM results. Equation (9) is introduced as the basis of the Empirical approach of this study.

(9) $$\sigma_{PW}=\beta \sigma_{PW,i}$$

where $\sigma_{PW}$ is the stress at the partial penetration welded corner at the peak height of the elephant foot buckling, β is the factor to be found by regression analysis and $\sigma_{PW,i}$ is the initial calculated stress at the partial penetration welded corner by the procedure of Batikha et al. [18] and the guidelines of the European code EC3 [32].

### 4.2. Calculation of the Stress Components at the Weld Throat Plane

The stress components shown in Figure 15 are directly related to the formation of elephant foot buckling. However, there is currently no procedure to calculate the stresses associated with this type of buckling for CFSTs. Alternatively, a method to evaluate the stresses caused by elephant foot buckling for circular plates by Batikha et al. [18] is adopted for this study. Although the adopted procedure was mainly developed for cylindrical soil tanks, the outer steel tube of CFSTs is subjected to similar loading conditions, which in general guarantees having a solution that can be later modified to be used for rectangular CFSTs. The domain of the problem, the governing equations and the modifications proposed by this study are presented next.

#### 4.2.1. Problem Domain

**Figure 16:**
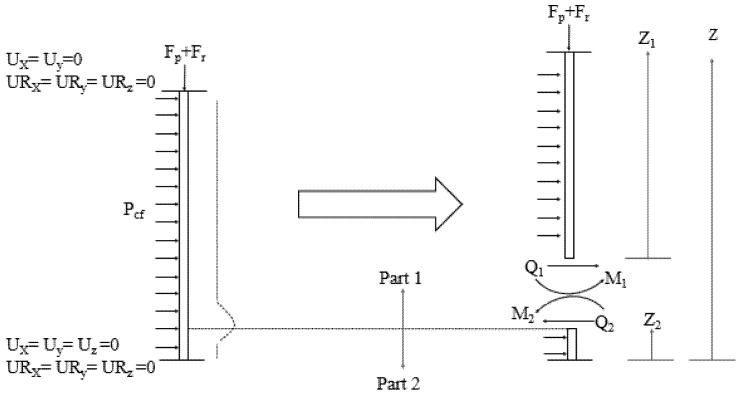
The domain of the elephant foot buckling problem in cylindrical plates.

Based on the procedure of Batikha et al. [18], the domain of the elephant foot buckling problem in cylindrical plates and subjected to vertical load and internal pressure is shown in Figure 16.

As shown in Figure 16, the domain of the problem is divided into two parts by taking a section cut just below the peak height of elephant foot buckling. In the reference study, the authors did not account for the boundary conditions at the far end, as the plate was long enough for the elephant foot buckling to not be influenced by them. However, this assumption may not be appropriate for shorter members like CFSTs, and these boundary conditions are considered in this study to determine this.

#### 4.2.2. Governing Equations

**Figure 17:**
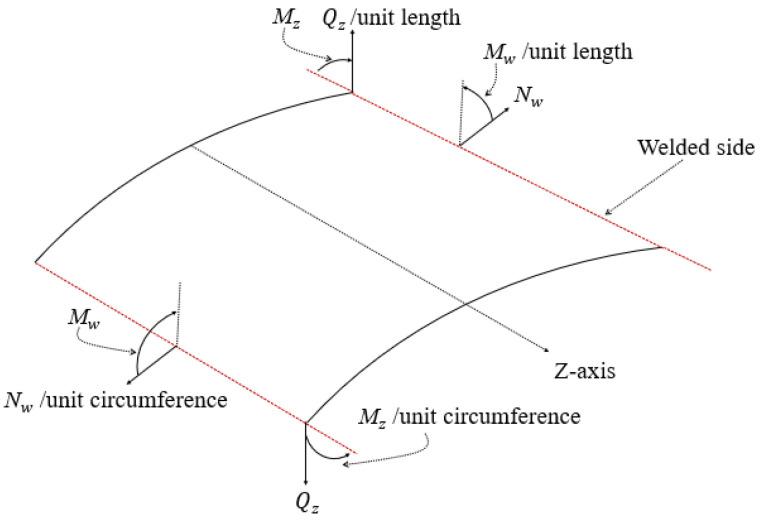
Stress and bending components of Equation (10).

The stress and bending components at any location of the problem shown in Figure 15 can be calculated from Equation (10).

(10a) $$N_{w}=\frac{E_{s}t}{r}w+v_{s}F\;\;$$

(10b) $$Q_{z}=-D\frac{d^{3}w}{dz^{3}}$$

(10c) $$M_{z}=-D\frac{d^{2}w}{dz^{2}}$$

(10d) $$M_{w}=v_{s}M_{z}$$

where $N_{w}$ is the hoop stress per unit length in the circumferential direction, $Q_{z}$ is the shear stress per unit length, $M_{z}$ is the meridional moment per unit circumference and $M_{w}$ is the circumferential moment per unit length. To give a better illustration, the stress and bending components are shown in Figure 17. F is the vertical force per unit circumference, $E_{s}$ is the Young’s modulus of steel, t is the thickness of the steel plate, r is the radius of the shell, w is the out of plane buckling deformation, $v_{s}$ is the Poisson’s ratio of steel and D is the flexural rigidity of the plate, calculated using Equation (11).

(11) $$D=\frac{E_{s}t^{3}}{12\left(1-v_{s}^{2}\right)}$$

Since all the stress and bending components are calculated in terms of the out of plane buckling, it is convenient to consider the maximum stress value at the peak of elephant foot buckling. The peak value of the elephant foot buckling can be calculated by solving the below differential equation [17,18].

(12) $$D\frac{d^{4}w}{dz^{4}}+\frac{E_{s}t}{r^{2}}w=P_{cf}+\frac{v_{s}F}{r}$$

where $P_{cf}$ is the internal pressure, which in this case is the confinement pressure. The solution presented by Batikha et al. [18] is also employed in this study. However, since the boundary conditions at the far end are considered in this case, the exponential function in the solution is replaced by a hyperbolic function, as recommended by Timoshenko and Krieger [17] and presented in Equation (13).

(13) $$\begin{array}{c} w=(c_{1}sin\frac{\pi z}{\lambda }sinh\frac{\pi z}{\lambda }+c_{2}sin\frac{\pi z}{\lambda }cosh\frac{\pi z}{\lambda }+c_{3}cos\frac{\pi z}{\lambda }sinh\frac{\pi z}{\lambda }\\ +c_{4}cos\frac{\pi z}{\lambda }cosh\frac{\pi z}{\lambda }+1)w_{m} \end{array}$$

where λ is the buckling half wavelength and $w_{m}$ is the membrane theory normal deflection, as presented in Equations (14) and (15), respectively.

(14) $$\lambda =\frac{\pi }{{\left(3\left(1-v_{s}^{2}\right)\right)}^{1/4}}\sqrt{rt}$$

(15) $$w_{m}=\left(P_{cf}+\frac{v_{s}F}{r}\right)\frac{r^{2}}{E_{s}t}$$

For proceeding, the coefficients appearing in Equation (13) have to be calculated with the aid of the hydrostatic load and internal confinement pressure, and thereafter the plated section has to be transformed to an equivalent circular section, where the stress resultants will finally be calculated.

#### 4.2.3. Transformation to Circular Cross-Section

Equations (10), (12) and (15) require the vertical load to be calculated per unit circumference of the shell, which also includes the value of the compressive residual stresses calculated as explained earlier in Section 2 of this paper. The internal confinement pressure is calculated from Equation (5), considering the actual load of the rectangular cross-section. In other trials, the confinement pressure was calculated for the transformed circular cross-section, but these were found to overestimate the internal pressure severely in smaller cross-sections, making it harder to fit the data in the regression analysis performed later.

The section transformation criterion followed is based on maintaining the same cross-sectional capacity for both sections, which means that both of them must have the same cross-sectional area. On this basis, Equation (16a–c) is presented.

(16a) $$A_{s,r}=A_{s,c}$$

(16b) $$A_{c,r}=A_{c,c}$$

(16c) $$r_{o,c}=\sqrt{\frac{A_{c,r}+A_{s,r}}{\pi }}$$

where $A_{s,r}$ is the area of steel in the rectangular cross-section, $A_{s,c}$ is the area of steel in the circular cross-section, $A_{c,r}$ is the area of concrete in the rectangular cross-section, $A_{c,c}$ is the area of concrete in the circular cross-section and $r_{o,c}$ is the outer diameter of the transformed circular cross-section. After calculating the outer diameter of the transformed section, it is possible to calculate the new equivalent thickness of the steel plate by deducting the concrete diameter, which can be calculated from the concrete area, from the calculated outer diameter. To clarify any confusion regarding how the original and transformed sections are used, the two main steps are listed below:

1. The forces acting on the steel plate (load per unit circumference and confinement pressure) are first calculated for the original cross-section.
2. The rectangular section is transformed to an equivalent circular section based on Equation (16) and the calculated forces are applied on the transformed section; this step will involve the equivalent thickness of the transformed section and will yield stress components per unit length or circumference.

#### 4.2.4. Calculating the Coefficients of the Buckling Shape Equation

At this stage, the calculated loads can be applied on the transformed cross-section, and the stress components can be calculated in terms of the elephant foot buckling deformation. The buckling shape equation, Equation (13), requires enforcing the boundary conditions shown in Figure 16 to calculate its coefficients. The shape equation is divided into two parts, and the boundary conditions are enforced, maintaining continuity between the two parts. The procedure to establish the buckling equation and its derivatives for each part is shown in Appendix A, and Appendix B shows the procedure of enforcing the boundary conditions and the final yielded equations to solve for the coefficients. Finally, the matrix form of the yielded equations in Appendix B is presented in Equation (17).

(17) $$\left|\begin{array}{cccccc} 0 & 0 & 0 & A_{14} & A_{15} & A_{16}\\ A_{21} & 0 & 0 & 0 & A_{25} & A_{26}\\ 0 & A_{32} & A_{33} & 0 & A_{35} & A_{36}\\ 0 & A_{42} & A_{44} & 0 & A_{45} & A_{46}\\ A_{51} & A_{52} & A_{53} & A_{54} & 0 & 0\\ A_{61} & A_{62} & A_{63} & A_{64} & 0 & 0 \end{array}\right|\times \left(\begin{array}{c} c_{11}\\ c_{12}\\ c_{13}\\ c_{14}\\ c_{21}\\ c_{22} \end{array}\right)=\left(\begin{array}{c} B_{1}\\ B_{2}\\ B_{3}\\ B_{4}\\ B_{5}\\ B_{6} \end{array}\right)$$

where the coefficients of $\left[A\right]$ and $\left[B\right]$ are separately calculated and arranged in Appendix C of this paper.

#### 4.2.5. Calculation of the Stress and Bending Components

For calculating the values of the coefficients in $\left[c\right]$ reported in Equation (17), an initial value of the peak height of the elephant foot buckling $h_{f}$ has to be assumed. Based on the mathematical solution presented in Equation (13), regardless of the initial assumption and for fixed end supports, the function will always return a maximum value of the elephant foot buckling at height of λ from the near end support [18]. Buckling along the height of the cross-sections with different assumed initial locations of the section cut was calculated, and the results were confirmed to be the same.

The procedure explained in this section will also return the same value of buckling at any normalized column height $\left(h/\lambda \right)$ for different plate dimensions, and this can be justified by taking a deeper look at Equation (13). Since the initial location of the section cut is assumed to be a factor of λ, λ at the denominator will disappear, making the solution independent from its value and, hence, also independent from the plate dimensions.

Based on the preceding procedure, the peak value of the elephant foot buckling is found to be $1.043w_{m}$; this value is substituted back into Equation (10), and the stress and bending components are calculated for the transformed section as presented in Equation (18).

(18a) $$N_{w}=\frac{1.043bp_{cf}+0.6F}{t}\;\left(\mathrm{MPa}\right)$$

(18b) $$Q_{z}=-0.016{\left(\frac{\pi }{\lambda }\right)}^{3}bt\left(bp_{cf}+0.3F\right)\left(\mathrm{MPa}\right)$$

(18c) $$M_{z}=0.008{\left(\frac{\pi }{\lambda }\right)}^{2}bt^{2}\left(bp_{cf}+0.3F\right)$$

(18d) $$M_{w}=0.0024{\left(\frac{\pi }{\lambda }\right)}^{2}bt^{2}\left(bp_{cf}+0.3F\right)$$

where a value of 0.3 for the Poisson’s ratio of steel is considered and $b=\sqrt{(A_{c,r}+A_{s,r})/\pi }$. The bending moment components $M_{z}$ and $M_{w}$ are calculated per unit length of the plate dimension, as shown in Figure 17, and to calculate the stress caused by these components, the calculated value has to first be multiplied by the plate dimension and then divided over the plate section modulus.

### 4.3. Substituting the Stress and Bending Components in EC3 Resultant Stress Formula

**Figure 18:**
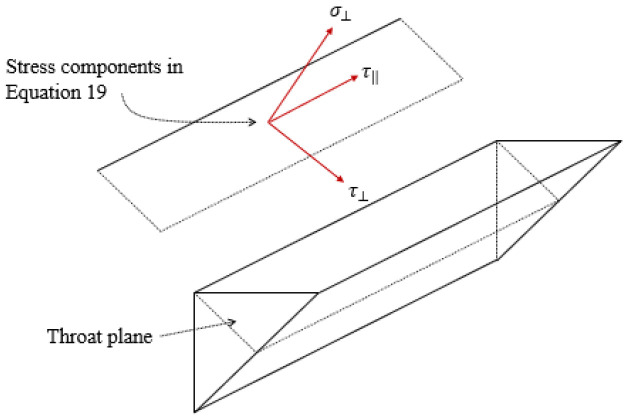
Stress components of Equation (19).

The method presented in the European code EC3 [32] to evaluate the stress at fillet welded connections is adopted for this study because it provides a lower bound solution based on the von Mises yield criterion, as presented in Equation (19).

(19) $$\sigma_{EC3}={\left[\sigma_{⊥}^{2}+3\left(\tau_{⊥}^{2}+\tau_{\|}^{2}\right)\right]}^{0.5}$$

where $\sigma_{EC3}$ is the resultant stress at the weld throat plane, $\sigma_{⊥}$ is the normal stress perpendicular to the weld throat plane, $\tau_{⊥}$ is the shear stress perpendicular to the weld throat plane and $\tau_{\|}$ is the shear stress parallel to the weld throat plane. The stress components reported in Equation (19) are also shown in Figure 18.

**Figure 19:**
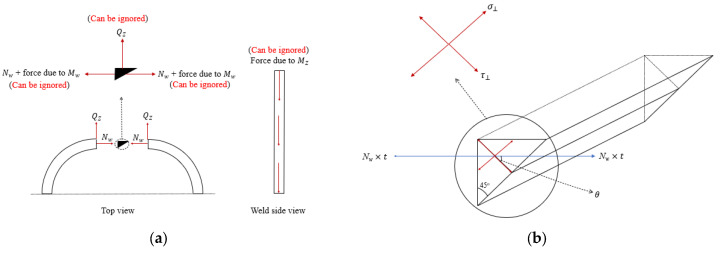
Stresses at the welded corner and the weld throat plane. (**a**) Reactions at the welded joint; (**b**) Reactions at the weld throat plane.

As explained earlier, the calculated stress and bending components have to be then transferred to the weld throat plane; this is carried out as shown in Figure 19.

**Figure 20:**
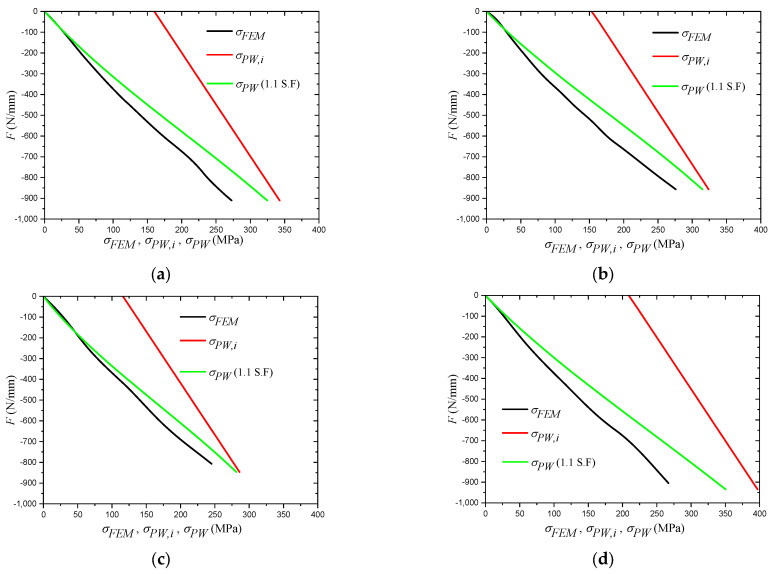
Influence of The Applied Vertical Load. (**a**) Sample 1; (**b**) Sample 2; (**c**) Sample 3; (**d**) Sample 4.

**Figure 21:**
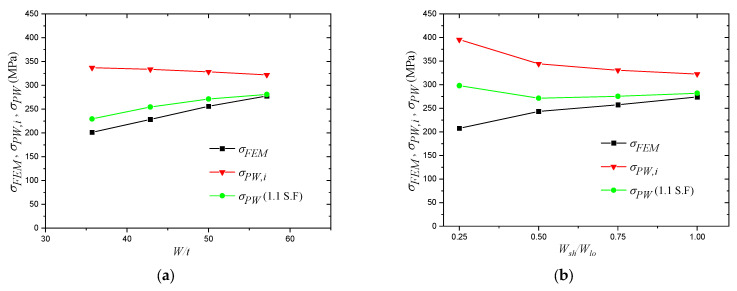

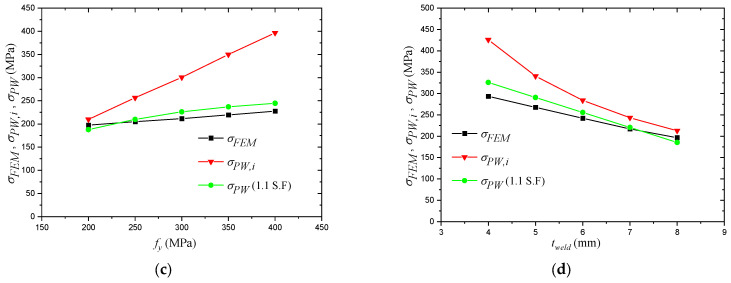
Parameters with significant influence on the resultant stress of the welded corner. (**a**) Influence of the plate width to thickness ratio (W/t); (**b**) Influence of the short plate to long plate ratio ($W_{sh}/W_{lo}$ ); (**c**) Influence of the yield strength of steel ($f_{y}$); (**d**) Influence of the weld throat thickness ($t_{weld}$ ).

**Figure 22:**
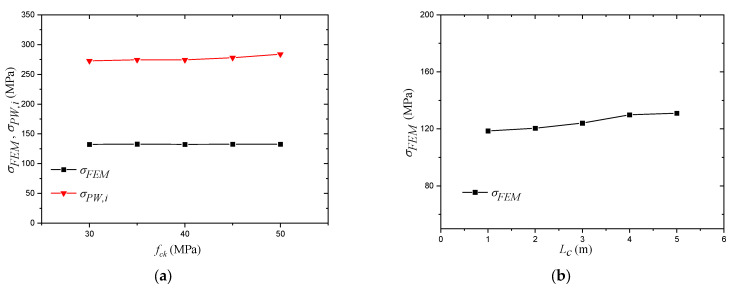
Parameters with small influence on the resultant stress of the welded corner. (**a**) Influence of concrete compressive strength ($f_{ck}$); (**b**) Influence of the column height ($L_{c}$).

The stress caused by the bending components $M_{z}$ and $M_{w}$ and the shear component $Q_{z}$ is found to have an extremely small value compared with the hoop stress component $N_{w}$. Taking a deeper look at Equation (10), these two components are always related to the second and third derivatives of the buckling shape equation; hence, unless the elephant foot buckling value is significantly large, these values will always remain small and can be safely ignored; on this basis, $\tau_{\|}$ component can be ignored. The formula to initially predict the stress at the welded corner is derived from Equation (19), as presented in Equation (20).

(20) $$\sigma_{PW,i}={\left[\sigma_{⊥}^{2}+3\tau_{⊥}^{2}\right]}^{0.5}$$

where the perpendicular and shear stress components can be calculated using Equation (21).

(21a) $$\sigma_{⊥}=\frac{(N_{w}\times t)\;sin\theta }{t_{weld}}$$

(21b) $$\tau_{⊥}=\frac{(N_{w}\times t)\;cos\theta }{t_{weld}}$$

where $t_{weld}$ is the thickness of the weld throat plane. At this stage, when the results obtained by Equation (20) are compared with the FEM results, sever overestimation by the equation is evident, as shown in Figure 20, Figure 21 and Figure 22, and, to overcome this issue, the results of the equation are fitted with the FEM results by means of regression analysis based on a large-scale parametric analysis covering most of the common engineering practices.

### 4.4. Parametric Analysis

A large-scale parametric analysis is performed by modelling a large number of single-cell rectangular columns with the parameters range, as shown in Table 6. The below discussion covers the influence of these parameters, at which the weld stress is calculated based on Equation (20) and plotted with the FEM results to find which parameters will create a significant difference between both results if changed. Some of the parameters shown in Table 6 are not variables in the proposed equation; therefore, their influence on the weld stress is only plotted considering the results of the FEM model to check if including them in the equation is necessary.

#### 4.4.1. Most Influencing Parameters

##### Influence of Vertical Load (F)

Figure 20 shows the influence of the applied vertical load on the weld stress obtained by the FEM and Equation (20). For this figure, sample 1 has the following parameters: $W_{lo}=600$ mm; $W_{sh}/W_{lo}=0.75$; t=14 mm; $t_{weld}=5$ mm; $L_{c}=1500$ m; $f_{ck}=35$ MPa and $f_{y}=300$ MPa; sample 2 has the same parameters as sample 1 except $W_{sh}/W_{lo}=1$; sample 3 and sample 4 have the same parameters as sample 1 except the material properties; for sample 3, $f_{ck}=35$ MPa and $f_{y}=250$ MPa; and for sample 4, $f_{ck}=35$ MPa and $f_{y}=350$ MPa.

Figure 20 shows significant overestimation by the initial proposed equation. Under low load levels, the difference between both estimations is the largest, and this can be explained keeping in mind that the initial equation assumes the same level of confinement pressure regardless of the applied vertical load because the formula to calculate this pressure is purely dependent on the material properties. The difference starts decreasing as the confinement pressure calculated by the FEM increases, and it reaches its minimum value at failure load. Taking sample 1 as an example, the FEM weld stress increased from 0 MPa to 272.6 MPa, while the stress calculated by the initial equation increased from 160.1 MPa to 342.8 MPa, i.e., the difference between both trends is 49.2%.

##### Influence of the Plate Width to Thickness Ratio (W/t)

Most of the modelled samples were rectangular samples. However, since the wider plate will always have higher elephant foot buckling, it is the one considered for this comparison. The influence of the ratio between the short and the long plates is discussed in the next subsection.

Figure 21a shows the influence of the long plate width to thickness ratio on the weld stress obtained by the FEM and Equation (20). For this figure, F=800 N/mm, $W_{sh}/W_{lo}=0.5$, t=14 mm, $t_{weld}=5$ mm, $L_{c}=1500$ mm, $f_{ck}=35$ MPa and $f_{y}=300$ MPa.

As shown in Figure 21a, the influence of the long plate width to thickness ratio on the results obtained by the FEM model is in clear agreement with what was observed experimentally. However, the equation results are unable to account for this parameter. The results obtained by the equation experience slight declination at high plate width to thickness ratio because the equation seems to be more influenced by the decrease in the confinement pressure than the increase in the elephant foot buckling deformation. For the FEM results, the stress at the welded corner increased from 201.3 MPa to 277.7 MPa, i.e., the difference is 38.0%. The results obtained by the initial equation experienced a decrease from 337.0 MPa to 322.1 MPa, i.e., the difference is 4.6%.

##### Influence of Short Plate to Long Plate Ratio ($W_{sh}/W_{lo}$)

Figure 21b shows the influence of the short plate to long plate ratio on the weld stress obtained by the FEM and Equation (20). For this figure, F=850 N/mm, $W_{lo}=600$ mm, t=14 mm, $t_{weld}=5$ mm, $L_{c}=1500$ mm, $f_{ck}=35$ MPa and $f_{y}=300$ MPa.

The change of the short plate to long plate ratio represents the change in the cross-section from a rectangular shape with a very small width of the short plate to a square shape with both plates having equal widths. Since the original rectangular or square shape was transformed to a circular shape, the influence of this parameter on the performance of the equation has to be concluded. Similar to the previous parameters, the ability of the equation to capture the influence of this parameter contradicts the FEM results. The FEM results experienced an increase from 207.5 MPa to 274.0 MPa, i.e., the difference is 32.0%, while the equation results experienced a decrease from 395.4 MPa to 322.5 MPa, i.e., the difference is 22.6%.

##### Influence of the Yield Strength of Steel ($f_{y}$)

Figure 21c shows the influence of the yield strength of steel on the weld stress obtained by the FEM and Equation (20). For this figure, F=700 N/mm, $W_{lo}=600$ mm, $W_{sh}/W_{lo}=0.75$, t=14 mm, $t_{weld}=5$ mm, $L_{c}=1500$ mm and $f_{ck}=35$ MPa.

Although both results experienced an increase of the stress by increasing the steel yield strength, the increase of the results calculated by the initial equation is much sharper than the FEM results. This difference is expected to be due to the earlier performed shape transformation. The FEM results experienced an increase from 197.4 MPa to 227.7 MPa, i.e., the difference is 15.3%, while the initial equation experienced an increase from 209.9 MPa to 396.6 MPa, i.e., the difference is 88.9%.

##### Influence of the Weld Throat Thickness ($t_{weld}$)

Figure 21d shows the influence of the weld throat thickness on the weld stress obtained by the FEM and Equation (20). For this figure, F=900 N/mm, $W_{lo}=600$ mm, $W_{sh}/W_{lo}=0.75$, t=14 mm, $L_{c}=1500$ mm, $f_{ck}=35$ MPa and $f_{y}=300$ MPa.

Similar to the steel yield strength, the same trend is showing with a much sharper decrease in the results obtained by the initial equation. The FEM results experienced a decreased from 293.3 MPa to 196.7 MPa, i.e., the difference is 49.1%, while the initial equation results experienced a decrease from 425.9 MPa to 212.9 MPa, i.e., the difference is 100%.

#### 4.4.2. Less Influencing Parameters

Other parameters are concluded to have a slight influence on the stress at the welded corner; these parameters are plotted in Figure 22. The column height is not a variable in the proposed equation and has a slight influence on the FEM results, while the concrete compressive strength does not have a significant influence on the difference between both results. The influence of these parameters will be ignored while performing the regression analysis.

### 4.5. Regression Analysis

The most influencing parameters on the proposed equation to estimate the stress at the welded corner were evaluated based on a large-scale parametric analysis. The most influencing parameters were found to be: the vertical applied load F, the plate width to thickness ratio W/t, the short plat to long plate ratio $W_{sh}/W_{lo}$, the yield strength of steel $f_{y}$ and the weld throat thickness $t_{weld}$. The plate width to thickness ratio and the short plate to long plate ratio are not independent from each other, and a new parameter that can combine both of them is necessary. To combine both parameters, the equivalent plate width is calculated using Equation (22), and a new parameter $W_{eq}/t$ is introduced. Several trials of regression analysis were performed in order to evaluate the need of including each of the most influencing parameters in the new form of the equation. However, excluding any of them severely influenced the final results. Such cases require performing multiple regression analysis, which would further complicate the case, or finding a suitable formula to combine the most influencing parameters, which is achieved using the dimensional factor α presented in Equation (23).

(22) $$W_{eq}=\sqrt{\left(W_{sh}^{2}+W_{lo}^{2}\right)/2}$$

(23) $$\alpha =\frac{FW_{eq}t_{weld}}{f_{y}\;\;t}\;\left({\mathrm{mm}}^{2}\right)$$

The derivation of factor α is based on how each of the parameters influenced the equation results when plotted with the FEM results. After a series of trials and errors to reach the most suitable formula, the best results were obtained when the parameters that caused a decrease in the calculated stress value by the initial equation when increased are kept as the numerator of the formula, i.e., F, $W_{eq}/t$ and $t_{weld}$, as shown in Figure 21a,b,d, respectively. The only parameter that caused an increase in the stress when increased, i.e., $f_{y}$, as shown in Figure 21c, is kept as the denominator of the formula. The calculated values of this factor for the performed analysis ranged between 17 mm^2^ and 800 mm^2^.

**Figure 23:**
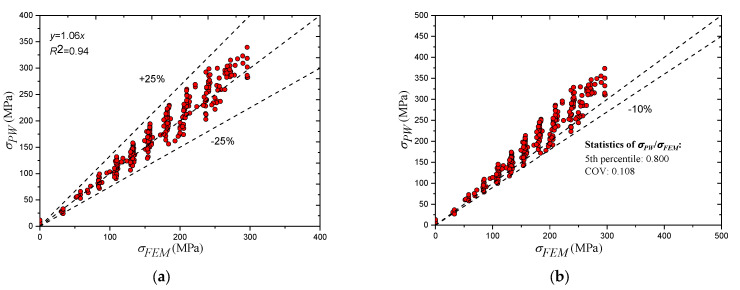

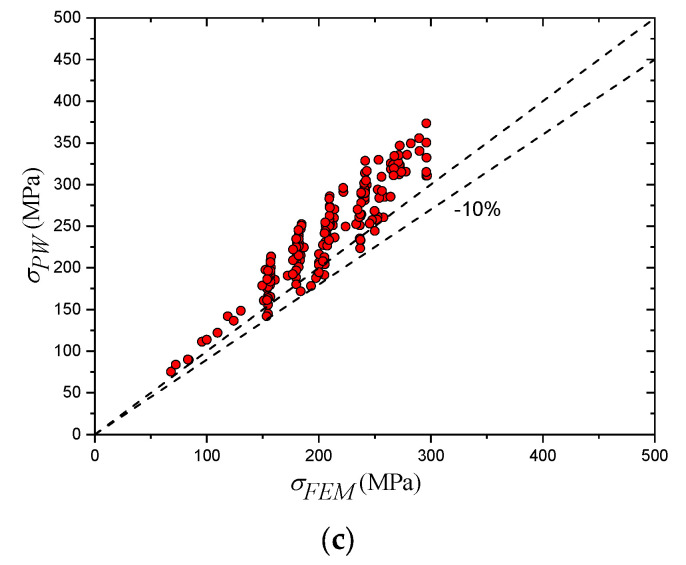
Proposed equation against FEM results. (**a**) Equation vs. FEM (no safety factor); (**b**) Equation vs. FEM (1.10 safety factor); (**c**) Equation vs. FEM (applied load above 60% of cross-sectional capacity).

The fitting of the equation results with the FEM results is based on Equation (24), and after trying several functions, the best results were obtained using a second-degree polynomial. The final formula to calculate the stress at the partial penetration welded corner is presented in Equation (25). The results of Equation (25) are plotted against the FEM results for a large number of points considering the range of parameters reported in Table 6, as shown in Figure 23a. It is worth mentioning that since the intention of the proposed equation was to yield a lower bound solution for most of the cases, some calculation points were excluded while performing the regression analysis. The reason behind excluding these points is because of the influence they had on the final equation, causing it to yield unacceptable underestimations for other points. However, excluding these points had no influence on the capability of the equation to yield a lower bound solution, as it only caused these points to be positioned higher than the +25% threshold shown in Figure 23a. An example of such points is when the short plate to long plate ratio is very small, namely, $W_{sh}/W_{lo}=0.25$; the overestimation of this case, which is the maximum calculated, is 43.6%.

(24) $$\frac{\sigma_{FEM}}{\sigma_{PW,i}}=f\left(\alpha \right)$$

(25) $$\sigma_{PW}=\beta \sigma_{PW,i}=\left(2.30\times 10^{-3}\alpha -1.41\times 10^{-6}\alpha^{2}\right)\sigma_{PW,i}$$

Since deriving Equation (25) involved a series of relatively complicated steps based on an empirical approach, the safety of the proposed equation should be strictly controlled. Two requirements are set to guarantee the safety of the proposed equation; the first one is maintaining a lower bound solution for at least 95% of the simulated cases, and the second one is preventing underestimation by more than 10%. Evaluation of the two requirements is performed considering a 1.1 safety factor; the calculated 5th percentile of the entire population of cases is less than 1, which satisfies the first requirement. The satisfaction of the second requirement is shown in Figure 23b, which shows that the entire population of cases lies above the 10% under the estimation line. Equation (25) is revised as presented in Equation (26). Furthermore, as previously concluded in the experimental part of this study, the performance of the partial penetration weld is most critical at higher load levels. To verify the performance of the equation in this case, Figure 23c is plotted. The figure clearly shows the satisfaction of the requirements when the applied load is above 60% of its cross-sectional capacity.

(26) $$\sigma_{PW}=1.1\;\beta \sigma_{PW,i}=1.1\left(2.30\times 10^{-3}\alpha -1.41\times 10^{-6}\alpha^{2}\right)\sigma_{PW,i}$$

### 4.6. Discussion

For a better illustration, the results obtained from Equation (26) are compared with the FEM results shown in Figure 20 and Figure 21. Figure 20 shows the performance of Equation (26) with different levels of the applied vertical load. The figure clearly demonstrates the ability of the equation to return a lower bound solution, especially under higher load levels. The figure also shows the capability of the equation to capture the FEM pattern after the performed regression analysis. Figure 21 shows the performance of Equation (26) when the most influencing parameters are changed based on the earlier performed parametric study. As shown in the figure, the equation only returned an underestimation for two cases: $f_{y}=200$ MPa and $t_{weld}=8$ mm. However, these underestimations never exceeded the 10% threshold. Moreover, the patterns of the equation are in good agreement with the FEM patterns, which supports its adequacy to cover the entire range of the considered parameters.

## 5. Conclusions

The behavior of welded multicell irregular shaped CFST specimens under different load conditions, different parameters and with the implementation of partial penetration weld at different locations was investigated. The feasibility of using partial penetration weld was verified, and the main conclusions of this study are listed below:

1. Using partial penetration weld for internal connections has no influence on the stiffness nor the ultimate capacity of the cross-section. However, when used for external corner connections, the influence on the stiffness of the column becomes more prominent, and tearing of the partial penetration welded corner near the location of elephant foot local buckling is possible, which indicates a necessity to evaluate the performance of this weld detail when used at this location.
2. The behavior of the Partial penetration welded corner is mostly influenced by the plate width to thickness ratio, and for smaller width to thickness ratios, the use of partial penetration weld for external corner connections becomes more feasible.
3. An equation to calculate the stress at the partial penetration welded corner was proposed based on the presented empirical procedure and with respect to the most influencing parameters. The safety of the proposed equation was strictly controlled, which guarantees a lower bound solution for at least 95% of the considered parameters range, with the stress in none of the cases being underestimated by more than 10%.

## Figures and Tables

**Table 1:** Dimensions of test specimens.

Specimen	a (mm)	b (mm)	c (mm)	d (mm)	e (mm)	f (mm)	g (mm)
PEN1	680	283	586	255	194	180	-
PEN2	680	283	586	255	194	180	-
PEN3	680	283	586	255	194	180	-
PEN4	680	283	586	255	194	180	-
PEN5	680	134	329	120	941	170	50
PEN6	680	134	329	120	941	170	50

**Table 2:** Steel properties.

Sample	Thickness (mm)	f_y_ (MPa)	f_u_ (MPa)	Elongation (%)
S-1	14	319.71	472.39	41.11
S-2	14	295.91	457.80	38.89
S-3	14	297.54	458.33	36.67
S-4	14	299.28	464.23	38.89
S-5	14	272.16	435.32	38.89
S-6	14	290.68	452.74	36.67
Average	14	295.88	456.80	38.52

**Table 3:** Concrete properties.

Mix Group	Concrete Class	Maximum Load (kN)	Compressive Strength (MPa)	Average (MPa)
PEN2 & PEN3	C35	984.64	43.76	41.48
		864.85	38.43	
		950.61	42.25	
PEN1 & PEN6	C35	779.90	34.66	34.35
		763.67	33.94	
		775.14	34.45	
PEN4 & PEN5	C35	958.11	42.58	43.48
		948.42	42.15	
		1028.53	45.71	

**Table 4:** Loading details.

Specimen	Load Type	Load Direction	Failure Load (kN)
PEN1	Cyclic	Vertical	13,992
PEN2	Monotonic	Slantwise	12,543
PEN3	Monotonic	Slantwise	11,980
PEN4	Cyclic	Slantwise	12,002
PEN5	Cyclic	Slantwise	16,023
PEN6	Cyclic	Slantwise	15,338

**Table 5:** Comparison between the FEM results and the experimental ones.

Specimen	FEM Ultimate Load (kN)	FEM Axial Displacement (mm)	Experiment Ultimate Load (kN)	Experiment Axial Displacement (mm)	Load Relative Error (%)	Displacement Relative Error (%)
PEN1	13,830	5.70	13,992	6.20	1.2	8.8
PEN3	12,023	4.20	11,980	4.50	0.4	7.1
PEN5	16,623	3.00	16,023	3.20	3.7	6.7
PEN6	15,519	3.00	15,338	3.00	1.2	0

**Table 6:** Parameters range.

Parameter	Range
Long plate width ($W_{lo}$)	300–1000 mm
Short plate width ($W_{sh}$)	150–500 mm
Plate thickness (t)	6–20 mm
Load (F)	0-section capacity
Weld throat thickness ($t_{weld}$)	4–8 mm
Column height ($L_{c}$)	1000–5000 mm
Concrete compressive strength ($f_{ck}$)	30–50 MPa
Steel yield strength ($f_{y}$)	200–400 MPa

## Data Availability

Data are contained within the article.

## Appendix A. Buckling Shape Equations for Part 1 and Part 2 in Figure 16

For part 1 Equation (13) is rewritten as:

(A1) $$\begin{array}{c} w_{1}=(c_{11}sinX_{1}sinhX_{1}+c_{12}sinX_{1}coshX_{1}+c_{13}cosX_{1}sinhX_{1}\\ +c_{14}cosX_{1}coshX_{1}+1)w_{m} \end{array}$$

At which, $X_{1}=\frac{\pi z_{1}}{\lambda }$. The first, second and third derivatives of the buckling shape equation are:

(A2) $$\begin{array}{cc} \frac{dw_{1}}{dz_{1}}=w_{m}\frac{\pi }{\lambda }[c_{11} & \left(cosX_{1}sinhX_{1}+sinX_{1}coshX_{1}\right)\\ & +c_{12}\left(sinX_{1}sinhX_{1}+cosX_{1}coshX_{1}\right)\\ & +c_{13}\left(cosX_{1}coshX_{1}-sinX_{1}sinhX_{1}\right)\\ & +c_{14}\left(cosX_{1}sinhX_{1}-sinX_{1}coshX_{1}\right) \end{array}$$

(A3) $$\begin{array}{cc} \frac{d^{2}w_{1}}{dz_{1}{}^{2}}=-2w_{m}{\left(\frac{\pi }{\lambda }\right)}^{2} & (-c_{11}cosX_{1}coshX_{1}-c_{12}cosX_{1}sinhX_{1}+c_{13}sinX_{1}coshX_{1}\\ & +c_{14}sinX_{1}sinhX_{1}) \end{array}$$

(A4) $$\begin{array}{cc} \frac{d^{3}w_{1}}{dz_{1}{}^{3}}=2w_{m}{\left(\frac{\pi }{\lambda }\right)}^{3} & [c_{11}\left(sinX_{1}coshX_{1}-cosX_{1}sinhX_{1}\right)\\ & +c_{12}\left(sinX_{1}sinhX_{1}-cosX_{1}coshX_{1}\right)\\ & +c_{13}\left(sinX_{1}sinhX_{1}+cosX_{1}coshX_{1}\right)\\ & +c_{14}\left(sinX_{1}coshX_{1}+cosX_{1}sinhX_{1}\right)] \end{array}$$

At the location of elephant foot buckling, $Z_{1}=0$, $X_{1}=0$; hence:

(A5) $$w_{1}=\;\left(c_{14}+1\right)w_{m}$$

(A6) $$\frac{dw_{1}}{dz_{1}}=w_{m}\frac{\pi }{\lambda }\left(c_{12}+c_{13}\right)$$

(A7) $$\frac{d^{2}w_{1}}{dz_{1}{}^{2}}=-2w_{m}{\left(\frac{\pi }{\lambda }\right)}^{2}\left(-c_{11}\right)$$

(A8) $$\frac{d^{3}w_{1}}{dz_{1}{}^{3}}=2w_{m}{\left(\frac{\pi }{\lambda }\right)}^{3}\left(-c_{12}+c_{13}\right)$$

At the far end support of part 1, the equation describing the deflection and its derivatives remains the same as reported in Equations (A1)–(A4). This location is at height $Z_{1}=Z-h_{f}$; hence, $X_{1}=\frac{\pi z_{1}}{\lambda }$, where $h_{f}$ is the assumed height of elephant foot buckling.

For part 2, Equation (13) is rewritten as:

(A9) $$\begin{array}{c} w_{2}=(c_{21}sinX_{2}sinhX_{2}+c_{22}sinX_{2}coshX_{2}+c_{23}cosX_{2}sinhX_{2}\\ +c_{24}cosX_{2}coshX_{2}+1)w_{m} \end{array}$$

At which $X_{2}=\frac{\pi z_{2}}{\lambda }$, while $Z_{2}=h_{f}$. The first, second and third derivatives of the buckling shape equations are:

(A10) $$\begin{array}{cc} \frac{dw_{2}}{dz_{2}}=w_{m}\frac{\pi }{\lambda }[c_{21} & \left(cosX_{2}sinhX_{2}+sinX_{2}coshX_{2}\right)\\ & +c_{22}\left(sinX_{2}sinhX_{2}+cosX_{2}coshX_{2}\right)\\ & +c_{23}\left(cosX_{2}coshX_{2}-sinX_{2}sinhX_{2}\right)\\ & +c_{24}\left(cosX_{2}sinhX_{2}-sinX_{2}coshX_{2}\right)] \end{array}$$

(A11) $$\begin{array}{cc} \frac{d^{2}w_{2}}{dz_{2}{}^{2}}=-2w_{m}{\left(\frac{\pi }{\lambda }\right)}^{2} & (-c_{21}cosX_{2}coshX_{2}-c_{22}cosX_{2}sinhX_{2}\\ & +c_{23}sinX_{2}coshX_{2}+c_{24}sinX_{2}sinhX_{2}) \end{array}$$

(A12) $$\begin{array}{cc} \frac{d^{3}w_{2}}{dz_{2}{}^{3}}=2w_{m}{\left(\frac{\pi }{\lambda }\right)}^{3} & [c_{21}\left(sinX_{2}coshX_{2}-cosX_{2}sinhX_{2}\right)\\ & +c_{22}\left(sinX_{2}sinhX_{2}-cosX_{2}coshX_{2}\right)\\ & +c_{23}\left(sinX_{2}sinhX_{2}+cosX_{2}coshX_{2}\right)\\ & +c_{24}\left(sinX_{2}coshX_{2}+cosX_{2}sinhX_{2}\right)] \end{array}$$

At the location of the near end support, $Z_{2}=0$ and $X_{2}=0$; hence:

(A13) $$w_{2}=\left(c_{24}+1\right)w_{m}$$

(A14) $$\frac{dw_{2}}{dz_{2}}=w_{m}\frac{\pi }{\lambda }\left(c_{22}+c_{23}\right)$$

(A15) $$\frac{d^{2}w_{2}}{dz_{2}{}^{2}}=-2w_{m}{\left(\frac{\pi }{\lambda }\right)}^{2}\left(-c_{21}\right)$$

(A16) $$\frac{d^{3}w_{2}}{dz_{2}{}^{3}}=2w_{m}{\left(\frac{\pi }{\lambda }\right)}^{3}\left(-c_{22}+c_{23}\right)$$

## Appendix B. Enforcing the Boundary Conditions in Figure 16

At the near end support, the boundary conditions are:

(A17) $$w_{2}=0$$

(A18) $$\frac{dw_{2}}{dz_{2}}=0$$

Enforcing these boundary conditions yields $c_{24}=-1$ and $c_{23}=-c_{22}$. At the location of the section cut between part 1 and part 2, the boundary conditions are:

(A19) $$M_{1}=M_{2}$$

(A20) $$Q_{1}=Q_{2}$$

(A21) $$w_{1}=w_{2}$$

(A22) $$\frac{dw_{1}}{dz_{1}}=\frac{dw_{2}}{dz_{2}}$$

while Q and M can be calculated from Equations (10b) and (10c), respectively. Enforcing the above boundary conditions yields:

(A23) $$c_{11}-c_{21}cosX_{2}coshX_{2}-c_{22}\left(cosX_{2}sinhX_{2}+sinX_{2}coshX_{2}\right)=sinX_{2}sinhX_{2}$$

(A24) $$\begin{array}{c} c_{12}-c_{13}+c_{21}\left(sinX_{2}coshX_{2}-cosX_{2}sinhX_{2}\right)+c_{22}\left(-2cosX_{2}coshX_{2}\right)\\ =sinX_{2}coshX_{2}+cosX_{2}sinhX_{2} \end{array}$$

(A25) $$-c_{14}+c_{21}sinX_{2}sinhX_{2}+c_{22}\left(sinX_{2}coshX_{2}-cosX_{2}sinhX_{2}\right)=cosX_{2}coshX_{2}$$

(A26) $$\begin{array}{c} -c_{12}-c_{13}+c_{21}\left(cosX_{2}sinhX_{2}+sinX_{2}coshX_{2}\right)+c_{22}\left(2sinX_{2}sinhX_{2}\right)\\ =cosX_{2}sinhX_{2}-sinX_{2}coshX_{2} \end{array}$$

At the far end support, the boundary conditions are:

(A27) $$w_{1}=0$$

(A28) $$\frac{dw_{1}}{dz_{1}}=0$$

Enforcing these boundary conditions yields:

(A29) $$\begin{array}{cc} c_{11}sinX_{1}sinhX_{1} & +c_{12}sinX_{1}coshX_{1}+c_{13}cosX_{1}sinhX_{1}+c_{14}cosX_{1}coshX_{1}\\ & =-1 \end{array}$$

(A30) $$\begin{array}{cc} c_{11}(cosX_{1}sinhX_{1} & +sinX_{1}coshX_{1})+c_{12}\left(sinX_{1}sinhX_{1}+cosX_{1}coshX_{1}\right)\\ & +c_{13}\left(cosX_{1}coshX_{1}-sinX_{1}sinhX_{1}\right)\\ & +c_{14}\left(cosX_{1}sinhX_{1}-sinX_{1}coshX_{1}\right)\;=0 \end{array}$$

## Appendix C. Coefficients of $\left[A\right]$ and $\left[B\right]$ in Equation (17)

Coefficients of $\left[A\right]$:

(A31) $$\left\{\begin{array}{c} A_{14}=-1\\ A_{15}=sinX_{2}sinhX_{2}\\ A_{16}=sinX_{2}coshX_{2}-cosX_{2}sinhX_{2} \end{array}\right.$$

(A32) $$\left\{\begin{array}{c} A_{21}=1\\ A_{25}=-cosX_{2}coshX_{2}\\ A_{26}=-\left(cosX_{2}sinhX_{2}+sinX_{2}coshX_{2}\right) \end{array}\right.$$

(A33) $$\left\{\begin{array}{c} A_{32}=1\\ A_{33}=-1\\ A_{35}=sinX_{2}coshX_{2}-cosX_{2}sinhX_{2}\\ A_{36}=-2cosX_{2}coshX_{2} \end{array}\right.$$

(A34) $$\left\{\begin{array}{c} A_{42}=-1\\ A_{43}=-1\\ A_{45}=cosX_{2}sinhX_{2}+sinX_{2}coshX_{2}\\ A_{46}=2sinX_{2}sinhX_{2} \end{array}\right.$$

(A35) $$\left\{\begin{array}{c} A_{51}=sinX_{1}sinhX_{1}\\ A_{52}=sinX_{1}coshX_{1}\\ A_{53}=cosX_{1}sinhX_{1}\\ A_{54}=cosX_{1}coshX_{1} \end{array}\right.$$

(A36) $$\left\{\begin{array}{c} A_{61}=cosX_{1}sinhX_{1}+sinX_{1}coshX_{1}\\ A_{62}=sinX_{1}sinhX_{1}+cosX_{1}coshX_{1}\\ A_{63}=cosX_{1}coshX_{1}-sinX_{1}sinhX_{1}\\ A_{64}=cosX_{1}sinhX_{1}-sinX_{1}coshX_{1} \end{array}\right.$$

Coefficients of $\left[B\right]$:

(A37) $$\left\{\begin{array}{c} B_{1}=cosX_{2}coshX_{2}\\ B_{2}=sinX_{2}sinhX_{2}\\ B_{3}=sinX_{2}coshX_{2}+cosX_{2}sinhX_{2}\\ B_{4}=cosX_{2}sinhX_{2}-sinX_{2}coshX_{2}\\ B_{5}=-1\\ B_{6}=0 \end{array}\right.$$
